# Effects of game-based learning and telesimulation methods on the knowledge, skills, motivations, and self-efficacy levels of nursing students in postpartum hemorrhage management: a randomized controlled trial

**DOI:** 10.1186/s12912-026-04878-4

**Published:** 2026-06-14

**Authors:** Yeşim Çetinkaya Şen, Gülten Güvenç

**Affiliations:** 1 Present Address: Department of Elderly Care, Haymana Vocational School, University of Ankara, Ankara, Türkiye; 2 Department of Obstetrics and Gynecology Nursing, Gulhane Faculty of Nursing, University of Health Sciences Turkey, Ankara, Türkiye; 3 Department of Elderly Care, Haymana Vocational School, University of Ankara, Ankara Üniversitesi Haymana Meslek Yüksekokulu, Çaldağ, Ankara Cd., Haymana/Ankara, 06860 Türkiye

**Keywords:** Nursing, Postpartum hemorrhage, Game-based learning, Telesimulation, Cognitive skills, Motivation, Self-efficacy

## Abstract

**Introduction:**

Recently, new methods such as game-based learning, mobile learning, and telesimulation have been used in lessons. In this study, we aimed to evaluate the effects of game-based learning and telesimulation methods on the knowledge, skills, motivation, and self-efficacy levels of nursing students in postpartum hemorrhage management.

**Methods:**

This randomized-controlled study comprised 177 nursing students who had attended an obstetrics and gynecology nursing course at a nursing department in Ankara, Türkiye. Participants were classified according to their grade point average and randomly assigned to four groups of equal size.

**Results:**

After training, the knowledge, skill, motivation, and self-efficacy scores in postpartum bleeding management were higher in the groups where telesimulation (Group II), Game-based learning (Group III), and both methods were used together (Group IV) compared with the control group (Group I, *p* < 0.001). Group IV scores were significantly higher than the other two experimental groups on knowledge (85.5 ± 9.3), skill (19.2 ± 6.3), motivation (141.2 ± 18.7), and self-efficacy (84.5 ± 7.9) scales (*p* < 0.001).

**Conclusion:**

Telesimulation and Game-based learning methods were more effective than virtual lecture for teaching postpartum hemorrhage management skills. However, during virtual lessons, combining telesimulation and Kahoot was more effective than all other methods.

**Trial registration:**

ClinicalTrials.gov ID, NCT05323903, Retrospectively registered. Registered on April 5, 2022.

**Supplementary Information:**

The online version contains supplementary material available at 10.1186/s12912-026-04878-4.

## Introduction

Postpartum hemorrhage (PPH) is a common obstetric emergency and a significant cause of maternal mortality globally [1]. Nurses are key team members on labor and delivery, and educating nursing students in PPH management is of most importance in recognition of and response to PPH Thus, it is critical to develop qualified nursing students with fundamental skills during their education [2, 3].

Nursing education provides theoretical training and the development of affective, cognitive, and psychomotor learning areas [4, 5]. Nowadays, the increase in the number of students, the physical inadequacies of laboratories, and the COVID-19 pandemic have brought distance education to the forefront. Innovative education methods are necessary for distance learning instead of traditional teaching methods used in conventional classroom settings, such as lectures, question-and-answer sessions, demonstrations, and in-person simulation. Methods that are not limited by time, place, or space constraints, can help to engage students’ interest and motivate them to learn and improve their clinical skills [4–8].

Telesimulation with standardized patients, one of the online methods used in medical, nursing, and other health professions education in recent years, increases students’ confidence and perceived competence in clinical skills [9, 10]. Telesimulation is a form of synchronous distance learning conducted via video conferencing [11]. Telesimulation allows for teaching critical thinking, decision making, communication, leadership, situational awareness, and application skills in a safe nursing education environment Alongside telesimulation, location- and time-independent web-based learning environments and mobile applications are increasingly being integrated into health professions education [12]. These digital platforms allow students to acquire, practice, and evaluate knowledge through mobile game–based learning activities, which have been shown to improve learning outcomes and enhance learner motivation [6, 13–16].

To the best of our knowledge, the combined use of telesimulation and gaming/web-based learning, including serious games, has not previously been examined in nursing education. Accordingly, this randomized controlled trial aimed to examine the effects of game-based learning, telesimulation, and their combined use on nursing students’ knowledge, clinical skills, motivation, and self-efficacy in the management of PPH.

## Methods

### Study design

This was a randomized controlled trial registered on ClinicalTrials.gov (NCT05323903/2022-04-05). Data were reported according to CONSORT 2010 protocol. The research adhered to the Consolidated Standards of Reporting Trials (CONSORT) guidelines to maintain methodological rigor and ensure complete transparency in reporting.

### Participants and sampling

In this study, no formal a priori sample size calculation was performed; instead, a census sampling approach was adopted. The study population comprised 180 nursing students who took an obstetrics and gynecology nursing course at a nursing department in Ankara, Türkiye, during the 2020–2021 academic year. Nursing students enrolled in the obstetrics and gynecology course in a nursing school in Ankara, were invited to participate in a study via email. A synchronous meeting was held on Microsoft Teams with the students who accepted the invitation. The students were informed about the study aims and were made aware that they could withdraw from the study at any time without any penalty or sanction. After completing the informed consent form, volunteering students were invited to join a WhatsApp group created by the researcher as a means of communication during the research process.

Inclusion criteria for the study consisted of volunteer students who were enrolled in the course for the first time, had a mobile phone, tablet or internet connection, were receiving distance education, had no prior nursing experience, and were receiving distance education during the COVID-19 pandemic. Students who did not complete telesimulation practice, participate in virtual lectures, or play games online were excluded.

### Randomization and group allocation

**Fig. 1:**
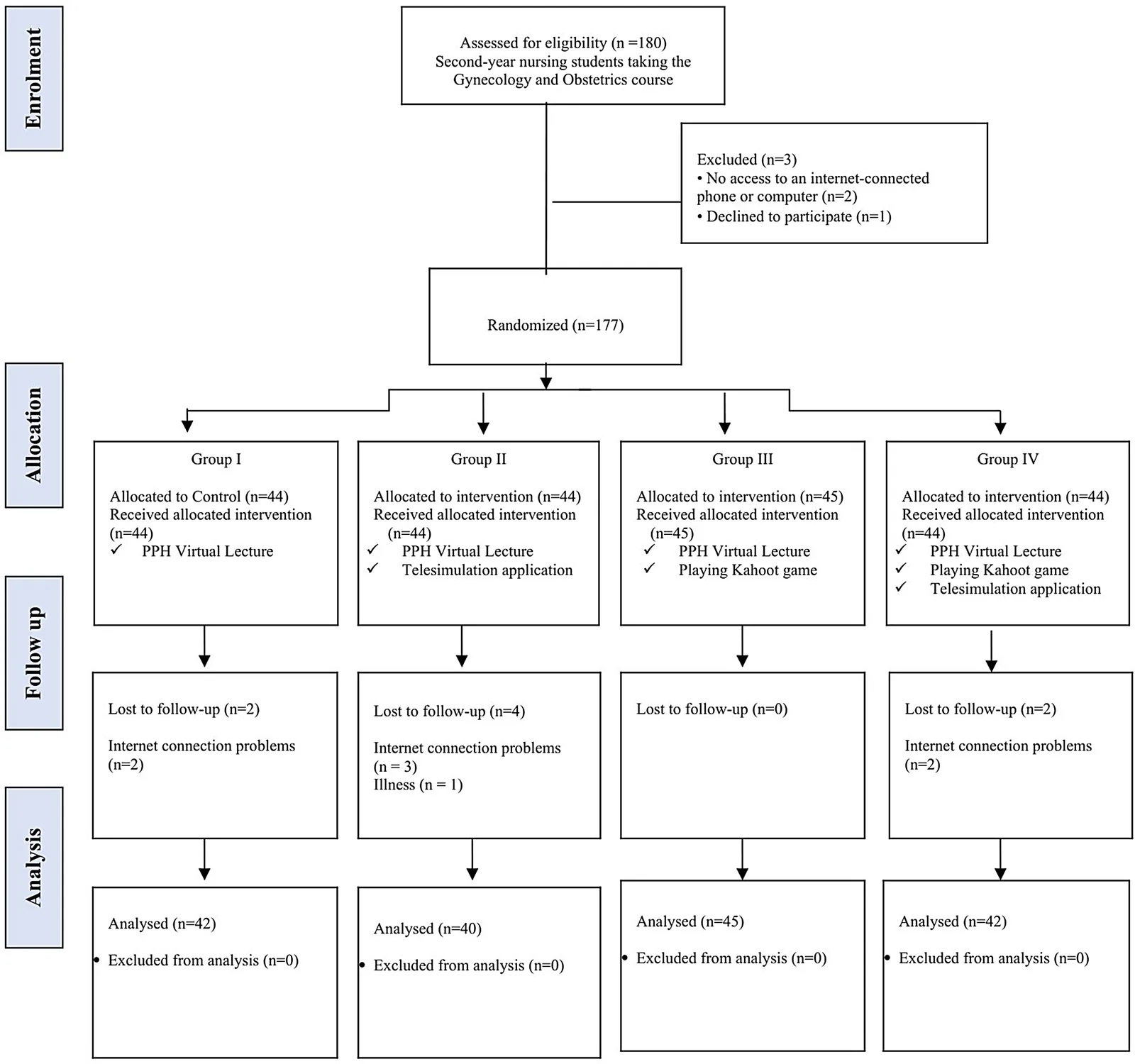
Flow diagram of nursing students’ inclusion in the study

The 177 nursing students who met the inclusion criteria were stratified according to cumulative grade point average and randomly allocated to one control group and three intervention groups using a block randomization method. All groups attended a virtual lecture. The study groups were defined as follows: Group I (virtual lecture only), Group II (virtual lecture plus telesimulation), Group III (virtual lecture plus game-based learning), and Group IV (virtual lecture combined with telesimulation and game-based learning). Following randomization, participants were allocated to separate Microsoft Teams classrooms: Group I (*n* = 44), Group II (*n* = 44), Group III (*n* = 45), and Group IV (*n* = 44) Additionally, Fig. 1 shows the flowchart of the process for including nursing students in the study.

### Study procedure

**Fig. 2:**
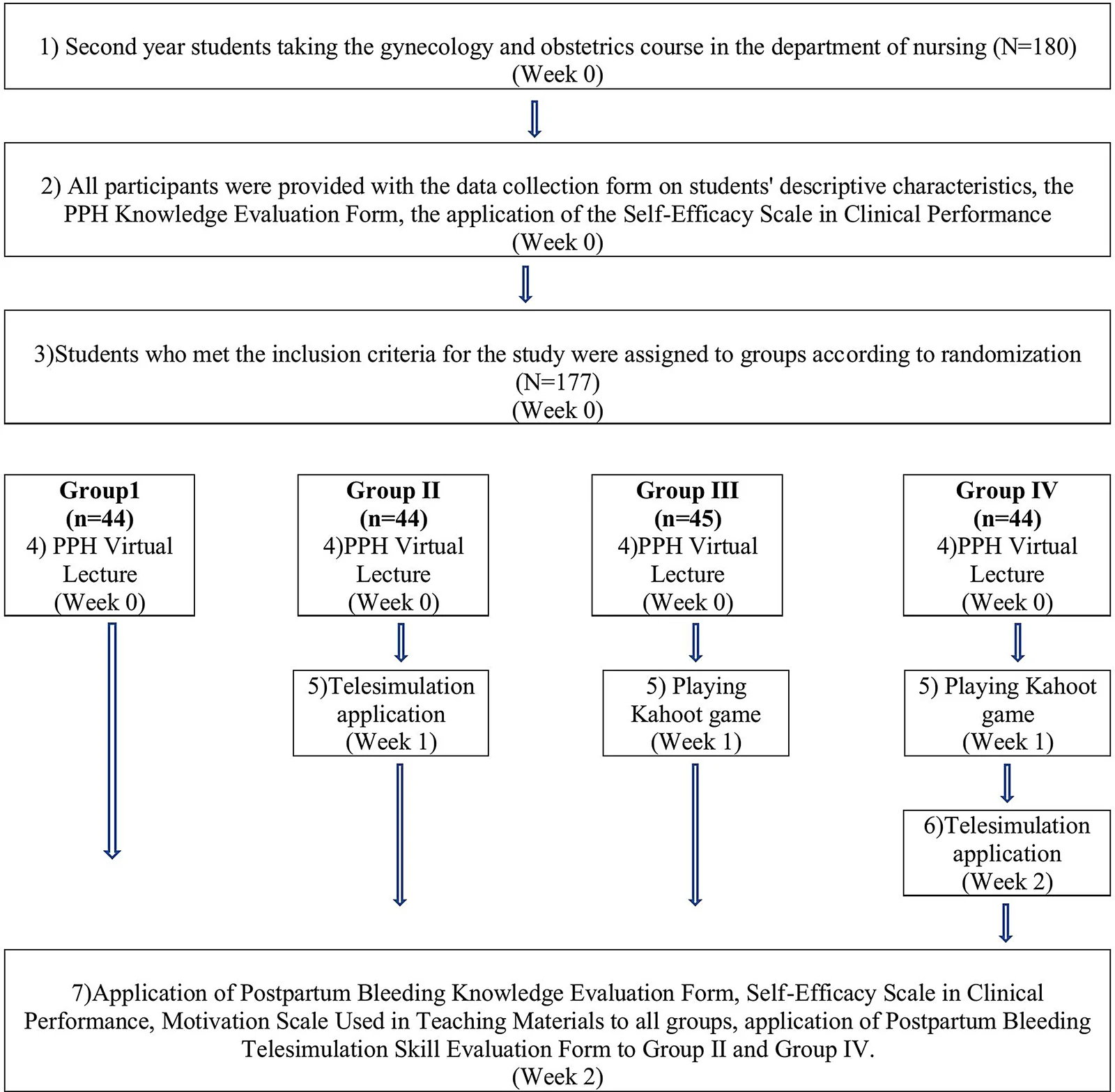
Study protocol for participants in a randomized controlled trial

The study was conducted in two stages, namely, pre-intervention and the intervention stages. The inclusion, randomization, and follow-up of participants in the randomized controlled trial were conducted as shown in Fig. 2.

### Pre-intervention phase

All participants completed a pre-test and attended a virtual lecture on postpartum hemorrhage (PPH) and its management, delivered via Microsoft Teams in two 40-minute sessions using Microsoft PowerPoint.

### İntervention phase

Within the scope of the research, all groups received the theoretical course on Postpartum Hemorrhage and Management on the same day, with the same instructor, the same content, and the same duration, using a synchronous distance learning method in accordance with the research protocol. This approach was chosen to ensure temporal and content equivalence in terms of initial knowledge levels among the groups. One week after the completion of the theoretical course, the Kahoot game-based learning intervention was initiated for Groups III and IV. Kahoot activities were implemented as a structured, multi-session learning process conducted on consecutive days, rather than a single session, to support the reinforcement and consolidation of theoretical knowledge. Following the completion of the theoretical course and Kahoot activities, a telesimulation intervention was conducted the following week for Groups II and IV. This allowed sufficient time for students to process and integrate the subject matter. To ensure educational equity, all educational interventions were made available to all study groups after completion of data collection.

### Game-based learning intervention

Among the various game-based programs used in education, Kahoot has recently become a popular online game-based learning platform that enables the use of smartphones, tablets, and computers without requiring complex controls and software [17–19]. Two researchers developed five tests, which were based on the learning objectives of the PPH training program, focusing on both cognitive skills and the postpartum hemorrhage checklist. These tests were created using the ‘Quiz’ feature of the Kahoot application, accessed through the ‘Create’ section on the website (www.kahoot.com). The tests were designed with multiple-choice questions or questions that require marking the correct answer, aligning with the cognitive and skills-based objectives of the training. After all groups had completed the virtual lecture, Groups III and IV participated in the Kahoot intervention. A 30-minute virtual meeting was held on Microsoft Teams, using Microsoft PowerPoint to explain game mechanics. A PIN code was sent to Group III and Group IV students via WhatsApp every day for 5 days. Students were given a time limit to complete each game after which the game was made inaccessible. Students spent a total of 20 min playing each game and were allowed to play the game only once. When students accessed the Kahoot screen for the first PIN, they were asked to define a nickname that would conceal their identity.

### Telesimulation intervention

The PPH telesimulation scenario was collaboratively developed by two researchers using the simulation design template proposed by Ünver and Başak (2016) [20]. It was implemented in accordance with simulation best practice standards of the International Nursing Association for Clinical Simulation and Learning (INACSL) [21]. In developing the telesimulation scenario, the learning objectives of the course were taken into consideration, as presented in Table 1. and the study scenario was revised based on feedback from two experts in obstetrics and gynecology nursing. Table 1. Postpartum hemorrhage learning objectives.

**Table 1:** Postpartum hemorrhage learning objectives

Domain	Learning objectives
Cognitive	Identify postpartum hemorrhage (PPH)
	List the risk factors for PPH
	Be able to perform a postpartum physical examination and distinguish signs of postpartum bleeding
	Knowledge of postpartum bleeding management principles and procedures
	Being able to list the symptoms seen in the mother due to bleeding
Skills	Emphasize the significance of applying correct care and understand the importance of a multidisciplinary approach to PPH
	Be able to monitor for changes in bleeding and taking relevant nursing precautions
	Establishing clear communication with the patient experiencing PPH and her family
Affective	Actively integrate into the environment in PPH management
	Actively communicate and collaborate with team members
	Empathize with of the patient and family having postpartum bleeding and be understanding, patient, and careful

After the Kahoot intervention, participants in Groups II and IV received telesimulation intervention. Before the telesimulation experience, the students were informed about the training expectations, the training video used in the telesimulation, and the scenario. During telesimulation, equipment such as webcams, smartphones, tablets, microphones, and headphones will be used and must be switched on. The learning objectives informed students about their roles and when to speak during the scenario. The timeline of the telesimulation was shared with the student and the preliminary information phase lasted approximately 5 min. The telesimulation program took 30 min in total. During this time, the facilitator used a skills assessment form to score student performance and guide debriefing. Telesimulation was planned and took into account INACSL 2021 best practice standards. A three-part video about postpartum bleeding was used. In the first part, the video was structured as the mother’s admission to the service after birth and their communication; in the second part, risk factors for PPH and physical examination were addressed; and in the last part, PPH management was explored. During the scenario, the patient monitors during the video and patient-related symptoms and findings were displayed to the students simultaneously. Groups of four people were formed with the students and the video was watched starting from the first chapter, with mini breaks (mini briefings) between each chapter. At the end of each section, the scenario was frozen with the pause button and the facilitator questioned each student about the information presented on the PPH skill assessment form in line with the scenario. The skill list comprises six main headings. For the first heading of the PPH skill list, ‘Patient Admission and Assessment,’ students were asked open-ended questions regarding their approach to the patient and the factors they would prioritize. Based on the students’ responses, the facilitator rated the corresponding practices in the skill list as either “Expressed” or “Did Not Express” After the interview with the first student, the audio was turned off, and the same procedure was repeated with the next student. This process continued until all six main headings had been assessed. The interview employed in the telesimulation was specifically developed for this study and has neither been used nor published elsewhere previously (Electronic supplementary material 1). Each group consisted of three or four students, and the interview duration for each student was approximately 14–15 minutes. To prevent the participants from hearing each other during the discussion, the sound system was turned off by the facilitator, and only the sound of the student who was given the right to speak was turned on. The PPH telesimulation skills evaluation form was completed while the student expressed the application according to the scenario. After these interventions, participants in all groups were asked to complete the post-test, which consisted of the knowledge of PPH management form and the self-efficacy in clinical performance scale.

### Data collection instruments

#### Data collection tools

Data were collected using a descriptive information form, a knowledge of PPH management form, a PPH telesimulation skills evaluation form, an instructional materials motivation survey (IMMS), and a self-efficacy in clinical performance scale (SECPS). Pretest measurements were obtained prior to the theoretical instruction, and posttest assessments were administered after completion of all interventions using a standardized time interval across all study groups.

### Descriptive information form

We developed a descriptive information form, which is a tool designed to gather basic information about the participants. The form consisted of 15 questions, each aimed at providing a comprehensive profile of the participants, including their age and educational background.

### Knowledge of PPH management form

The 25-item original form, developed by the researchers, was created based on a review of the literature and in consideration of the learning outcomes of the obstetrics and gynecology nursing course [1–3, 22]. After receiving expert opinions from three obstetrics and gynecology nursing experts who had previously worked on the subject that was not included in the study, we revised the form and conducted a pilot study on 52 nursing students on April 3, 2021. The pilot study was conducted with 52 nursing students who did not participate in the main study, and data obtained during the pilot phase were excluded from the final analyses. Indexes of difficulty and discrimination were calculated for each item, and five items with a discrimination index lower than 0.30 were excluded from the form. The revised form consisted of 20 items. Data from the pilot study excluded in the analysis. Each correct and wrong answer scored 5 and 0 points, respectively. The scores ranged from 0 to 100, with higher scores indicating a higher knowledge of PPH management. The Kuder–Richardson value of the knowledge evaluation form was 0.84. This scale provides an opportunity to measure how much knowledge the student has gained, which was one of the learning objectives (Electronic supplementary material 2).

### PPH telesimulation skills evaluation form

The PPH telesimulation skills evaluation form was developed by the researchers based on the literature [1–3]. The form included six categories: patient admission and evaluation, communication, physical examination, hypovolemic shock, PPH management, and blood transfusion. The form, consisting of 30 items, was revised based on the opinions of an expert in assessment and evaluation in nursing education and three experts with simulation studies in obstetrics and gynecology nursing. Participants were awarded one point if they were able to correctly express the expected steps for PPH, and zero points if they were unable to express them. Possible scores ranged from 0 to 30, with higher scores indicating better skills for PPH telesimulation. The telesimulation skill form was filled out during the telesimulation (Electronic supplementary material 3).

### Instructional materials motivation survey (IMMS)

Motivation Scale Used in Teaching Materials was developed by Keller et al. in 2006, advancing further from Keller’s (1987) ARCS model to understand motivation. The ARCS model is an acronym from attention, relevance, confidence, and satisfaction. and the survey comprised 36 items for these four subscales [23]. Items were scored on a 5-point Likert scale, ranging from 1 (definitely incorrect) to 5 (definitely correct). Possible scores ranged from 36 to 180, and the midpoint was 108. IMMS was translated into Turkish by Acar (2009). Cronbach’s alpha of the Turkish version of IMMS and our study were 0.92 and 0.85, respectively [24], (Electronic supplementary material 4).

### Self-efficacy in clinical performance scale (SECPS)

The SECPS scale was developed by Cheraghi et al. (2009) and translated into Turkish by Pozam and Zaybak (2015) [25, 26]. The scale has 37 items in four subscales: assessment, diagnosis and planning, implementation, and evaluation. There are 11 answers on the scale: 0%, 10%, 20%, 30%, 40%, 50%, 60%, 70%, 80%, 90%, 100%. The answer “I am not sure” corresponds to 0% and “I am completely sure” to 100%. Scores for each item ranged from 0 (not sure) to 100 (definitely sure), and the average was calculated to measure the overall SECPS score [26]. Cronbach’s alpha of the original and Turkish versions of SECPS were 0.96 and 0.98; in our study Cronbach’s alpha was 0.92. An understanding of student self-efficacy in clinical performance is an important step in determining the extent to which the learning objectives of nursing education programs have been achieved (Electronic supplementary material 5).

### Data analysis

SPSS version 25.0 was used for data analysis. In addition to descriptive statistics, which included frequency, percentage, mean, standard deviation, and minimum and maximum values, the Chi-squared test was used to compare qualitative data. Kolmogorov–Smirnov test, skewness and kurtosis, and graphical methods (histograms, Q-Q plots, stem and leaf plots, and boxplots) were used to test for normality. Quantitative data that met normal distribution were analyzed using independent samples T-test and One-Way ANOVA, while Tukey’s HSD test was used for multiple comparisons. A paired samples T-test was used for the pre-test–post-test comparison. Pearson’s correlation analysis was used to analyze the correlation between variables. The statistical significance level was set at *p* < 0.05.

## Results

The participants’ average age is 20.1 ± 0.9 years, and the majority (87.8%) are women. There was no significant difference between the intervention and control groups regarding age, gender, economic status, desire to be a nurse, and place of residence (*p* > 0.05). Some characteristics of the students in the intervention and control groups regarding their technology use were examined. No statistically significant differences were found between the groups in terms of participation in the simulation application, use of digital tools for course-related activities, playing digital games via devices such as mobile phones or computers, and prior awareness of Kahoot (*p* > 0.05).

No significant difference was found in the pre-test scores between all groups from the knowledge of PPH management form (*p* = 0.282). A significant difference was found between the groups after Kahoot and telesimulation interventions (*p* = 0.035). Multiple comparisons revealed that the highest and the lowest scores were obtained by Groups IV (85.5 ± 9.3) and I (74.6 ± 20.9), respectively. Intergroup comparison showed that post-test scores were significantly higher than pre-test scores for all groups (*p* < 0.001). In addition, the knowledge scores of the groups in which only telesimulation (Group II 80.7 ± 18.1) or Kahoot was used (80.0 ± 15.6) were significantly higher than those in the virtual lecture (Group I 74.6 ± 20.9).

Analysis of PPH telesimulation skills revealed that total PPH telesimulation skill scores significantly increased in Groups II and IV after the telesimulation intervention (*p* < 0.001). There was a significant difference between patient admission and evaluation (*p* = 0.018), communication (*p* = 0.003), physical examination (*p* < 0.001), PPH management (*p* < 0.001), and blood transfusion (*p* < 0.012). The intergroup comparison revealed that Group IV scored higher than Group II in all categories of PPH telesimulation skills. There was also a significant difference between the scores obtained by the two intervention groups from the PPH telesimulation skills form (*p* < 0.001) (Table 2).

**Table 2:** Comparison of PPH skill scores in Groups II and IV after telesimulation application

	Group II (*n* = 38)	Group IV (*n* = 39)	St.	*P*
Admission of postpartum woman	1.7 ± 1.2 2.0 (0.0–3.0)	2.3 ± 1.1 3.0 (0.0–3.0)	t=-2.413	0.018
Communication with postpartum woman	1.8 ± 1.2 2.0 (0.0–3.0)	2.5 ± 0.8 3.0 (0.0–3.0)	t=-3.101	**0.003**
Physical examination	1.5 ± 0.9 2.0 (0.0–3.0)	2.3 ± 0.8 2.0 (0.0–3.0)	t=-3.860	**0.000**
Hypovolemic shock	1.4 ± 1.5 1.0 (0.0–6.0)	2.0 ± 1.7 2.0 (0.0–6.0)	t=-1.678	0.098
PPH management	6.0 ± 3.3 6.0(0.0–13.0)	9.1 ± 3.2 10.0(1.0–17.0)	t=-4.188	**< 0.001**
Blood transfusion	0.5 ± 0.8 0.0(0.0-0.2)	1.0 ± 1.0 1.0(0.0–2.0)	t=-2.591	**0.012**
Postpartum hemorrhage skills total score	12.8 ± 6.3 11.0(1.0 ± 29.0)	19.2 ± 6.3 20.0(4.0–30.0)	t=-4.438	**< 0.001**

Group II: Virtual lecture +telesimulation. Group IV: Virtual lecture + Kahoot + telesimulation

The study’s findings indicated a discrepancy in the IMMS scores between Group IV and the other groups (*p* < 0.05). Furthermore, there was a significant difference between all subscales of IMMS, namely attention (*p* < 0.001), relevance (*p* < 0.001), confidence (*p* < 0.001), and satisfaction (*p* < 0.001) Compared with other groups, Group IV had the highest score from the IMMS (141.2 ± 18.7) and its subscales. Regarding scores from the IMMS, the motivation level of Group III (Kahoot) was higher than that of Group II (Table 3). Additionally, a distinction was observed between Group IV and Groups I and II in the SECPS post-test scores (*p* < 0.05). Group IV demonstrated the highest mean score (84.5 ± 7.9), in comparison to the other groups (Table 4).

**Table 3:** Intergroup comparison of IMMS scores

	Group I (*n* = 42)	Group II (*n* = 38)	Group III (*n* = 45)	Group IV (*n* = 39)	St.	P*	Diff
Attention	36.3 ± 6.8 37.0 (25.0–49.0)	36.3 ± 9.6 39.5 (10.0–50.0)	38.9 ± 5.9 39.0 (24.0–50.0)	43.4 ± 5.7 44.0 (32.0–50.0)	F = 8.796	< 0.001	1 and 3–4
Relevance	30.0 ± 4.7 30.0 (21.0–37.0)	30.4 ± 7.5 33.0 (8.0–40.0)	32.1 ± 4.1 33.0 (22.0–39.0)	35.3 ± 4.1 36.0 (26.0–40.0)	F = 8.449	**< 0.001**	4 and others
Confidence	31.4 ± 6.4 32.0 (22.0–45.0)	30.7 ± 7.4 30.5 (9.0–43.0)	32.0 ± 5.6 33.0 (18.0–44.0)	36.9 ± 5.5 38.0 (22.0–44.0)	F = 8.136	**< 0.001**	4 and others
Satisfaction	21.5 ± 5.1 21.5 (9.0–30.0)	21.8 ± 5.6 23.5 (6.0–30.0)	23.4 ± 4.1 24.0 (13.0–30.0)	25.5 ± 4.3 26.0 (12.0–30.0)	F = 5.850	**0.001**	4 and 1–2
Total	119.2 ± 20.4 122.0 (87.0–161.0)	119.2 ± 29.2 126.5 (33.0–161.0)	126.5 ± 18.3 130.0 (82.0–161.0)	141.2 ± 18.7 145.0 (95.0–164.0)	F = 8.854	**< 0.001**	4 and others

Group I: Virtual lecture, Group II: Virtual lecture + Telesimulation, Group III: Virtual lecture + Kahoot, Group IV: Virtual lecture + Kahoot + Telesimulation

**Table 4:** İntergroup comparison of SECP scores

		Group I (*n* = 42)	Group II (*n* = 38)	Group III (*n* = 45)	Group IV (*n* = 39)	St.	P*	Diff
Assessment	Pretest ^a^	74.5 ± 19.1 76.0 (11.0–100.0)	77.1 ± 16.1 79.0 (30.0–100.0)	77.1 ± 13.1 80.0 (44.0–100.0)	80.4 ± 13.3 81.0 (53.0–100.0)	F = 0.996	0.396	--
	Posttest ^b^	67.7 ± 10.4 70.0 (37.0–84.0)	80.1 ± 6.8 81.0 (65.0–94.0)	80.7 ± 10.2 82.0 (57.0–100.0)	83.4 ± 8.8 81.0 (63.0–100.0)	F = 23.963	**< 0.001**	1 and 2-3-4
	**F**	4.324	1.008	1.802	1.173			
	P**	**0.044**	0.322	0.186	0.286			
	**Diff**	c and a-b	--	--	--			
Diagnosis and planning	Pretest ^a^	66.1 ± 23.7 75.0 (8.0–99.0)	76.1 ± 16.8 77.0 (30.0–100.0)	74.1 ± 14.9 76.0 (41.0–100.0)	76.8 ± 13.2 74.0 (52.0–100.0)	F = 3.176	**0.026**	1 and 2–4
	Posttest ^b^	68.4 ± 11.7 71.5 (32.0–88.0)	80.2 ± 7.3 80.5 (69.0–98.0)	79.5 ± 11.3 79.0 (57.0–100.0)	83.5 ± 9.2 82.0 (61.0–100.0)	F = 17.379	**< 0.001**	1 and 2-3-4
	**F**	0.302	1.653	3.169	5.461			
	P**	0.586	0.207	0.082	**0.025**			
	**Diff**	--	--	--	a ile b-c			
Implementation	Pretest ^a^	77.1 ± 20.2 79.5 (9.0–100.0)	80.6 ± 16.6 81.0 (30.0–100.0)	82.4 ± 12.7 84.0 (41.0–99.0)	81.5 ± 14.3 83.0 (45.0–100.0)	F = 0.852	0.468	--
	Posttest ^b^	72.6 ± 9.8 74.0 (44.0–95.0)	84.5 ± 8.7 88.0 (64.0–96.0)	82.1 ± 11.1 86.0 (52.0–98.0)	86.0 ± 8.0 87.0 (74.0–100.0)	F = 16.178	**< 0.001**	1 and 2-3-4
	**F**	1.784	1.575	0.009	2.762			
	P**	0.189	0.217	0.925	0.105			
	**Diff**	--	--	--	--			
Evaluation	Pretest ^a^	73.4 ± 21.5 79.0 (17.0–100.0)	77.1 ± 17.3 78.0 (30.0–100.0)	76.8 ± 14.3 78.0 (42.0–100.0)	79.7 ± 12.5 80.0 (53.0–100.0)	F = 0.992	0.398	--
	Posttest ^b^	70.8 ± 9.9 72.5 (40.0–87.0)	82.7 ± 8.8 85.0 (60.0–93.0)	80.9 ± 13.9 85.0 (45.0–100.0)	85.1 ± 8.6 87.0 (70.0–100.0)	F = 14.114	**< 0.001**	1 and 2-3-4
	**F**	0.475	3.068	1.471	4.177			
	P**	0.495	0.088	0.232	**0.048**			
	**Diff**	--	--	--	a ile b-c			
Total	Pretest ^a^	73.5 ± 19.9 77.0 (11.0–100.0)	77.6 ± 16.4 77.5 (30.0–100.0)	77.7 ± 13.0 80.0 (42.0–99.0)	79.6 ± 12.9 80.0 (51.0–100.0)	F = 1.097	0.352	--
	Posttest ^b^	69.9 ± 9.7 72.8 (40.0–84.5)	81.9 ± 7.1 84.8 (65.0–95.0)	80.8 ± 10.3 81.8 (55.3–99.3)	84.5 ± 7.9 84.3 (68.0–100.0)	F = 21.495	**< 0.001**	1 and 2–4
	**F**	1.178	2.014	1.249	3.463			
	P**	0.284	0.164	0.270	0.071			
	**Diff**	--	--	--	--			

Group I: Virtual lecture, Group II: Telesimulation + Virtual lecture, Group III: Kahoot + Virtual lecture, Group IV: Virtual lecture + Kahoot + Telesimulation

## Discussion

PPH requires rapid diagnosis, careful monitoring and coordinated management as it can lead to maternal death. Therefore, it is important to improve theoretical knowledge and clinical skills by increasing the motivation of nursing students. This study aimed to analyze the effects of using different teaching methods (Virtual lecture, Kahoot, and telesimulation), separately and in combination, on the knowledge, skills, motivation, and self-efficacy levels of nursing students concerning PPH management.

Following the educational intervention, both PPH knowledge scores and IMMS and SECPS scores were observed to be higher in the other groups compared to the control group. The highest knowledge score was observed in Group IV, where the combination of Kahoot and telesimulation was employed, followed by the telesimulation and Kahoot group. Although no study in the literature uses telesimulation and game-based learning methods in PPH management training, studies on different subjects have shown that Kahoot and telesimulation are effective in improving knowledge. Studies using the Kahoot platform have shown that it significantly increases the knowledge of nursing students [19, 27]. In studies by Özaras Öz and Ordu (2021) assessing knowledge and skills in intramuscular injection in nursing students, knowledge scores were significantly higher after using Kahoot [19]. In our study, the motivation and SECPS score of the students who applied Kahoot were higher than those of the control group and the telesimulation group. This finding indicates that Kahoot can be considered a viable teaching method for teaching PPH to students. Published studies have reported that Kahoot is an engaging, interactive, entertaining platform that provides the opportunity to compete with other students, problem-solving skills, and enhances medical learning by increasing the level of attention and motivation of students [28–36] Chang et al. (2022) found that game-based learning and the watch–summarize–question strategy had a significant effect on learning achievement and the levels of engagement and self-efficacy in nursing during the COVID-19 pandemic [15]. Bayram and Çalışkan (2019) found the effects of game-based virtual reality use on psychomotor skill acquisition to be realistic, entertaining, and sufficient in theoretical and clinical aspects [37]. However, Ranieri et al. (2018) found that while Kahoot was more effective for theoretical subjects, it was less effective for practical skills [38].

In our study, the knowledge score of the telesimulation group was found to be higher than the control and Kahoot groups, while the motivation and SCEPS scores were lower than Kahoot and GroupIV. Looking at studies using telesimulation in the literature, Mileder et al. (2021) reported an increase in knowledge of neonatal resuscitation among medical students and nurses after telesimulation [39]. Similarly, Falcioni et al. (2022) found a significant increase in knowledge scores following telesimulation [40]. Liu et al. (2021) reported that participants using telesimulation for ultrasound-guided regional anesthesia in Ethiopia increased their mean total score from 51% at the pre-test to 84% post-test [41]. In some studies, on telesimulation, found that its effects on nursing skills was limited or no different than traditional simulation methods telesimulation might teach students or nurses clinical skills in patient education, counseling, communication, decision making, and clinical reasoning. Since developing complex psychomotor skills depends on exposure to hospital settings, telesimulation cannot be a complete substitute for clinical experience [42]. However, integrating mannequins and other technical equipment into telesimulation can facilitate advancing such skills beyond university and simulation lab settings.

The study revealed that Group IV, which utilized a combination of Kahoot and telesimulation, demonstrated significantly higher PPH knowledge, skill, IMMS, and SECPS scores than all other groups. However, no studies using Kahoot, telesimulation, or these methods together were found in the literature. It is thought that the combined use of Kahoot and telesimulation methods provides students with clear learning objectives, helps them to observe, compare, summarize, and manage learning tasks, structures their knowledge by stimulating feelings of curiosity and competition, and increases their motivation to learn. Others have reported that telesimulation is an effective learning method that integrates remote clinical practices and contributes to clinical performance, critical thinking, and self-efficacy in nursing students [43, 44]. Therefore, telesimulation and game-based learning methods could be integrated into the nursing curriculum, and further studies should be conducted on the effect of these methods on the clinical performances of nursing students in a larger population. In addition, using different learning materials together ensures that students are exposed to the subject regularly. In education, it has been shown that the use of spaced repetition and active learning methods increases the efficiency of learning a subject. Spaced learning encourages permanent learning by allowing students to review and reinforce information at regular intervals [45–47].

In our study, it was concluded that telesimulation and Kahoot were effective learning methods in developing nursing students’ knowledge, skills, motivation, clinical performance, and self-efficacy compared to theoretical education. When both methods were used together, students reinforced the subject by making spaced repetitions and learning efficiency increased. In light of these findings, it is recommended that plans for the integration of similar methods be considered in future studies.

### Limitations

First, due to the COVID-19 pandemic, the students received remote education, therefore, face-to-face learning was not evaluated and the participants encountered difficulties accessing the technological devices necessary for remote education. Second, the study only examined one subject of the gynecology and obstetrics nursing course. To further enhance the understanding of nursing skills, additional studies should be conducted on other topics. PPH telesimulation skills evaluation form and scoring have been developed by researchers. Validity and reliability analyses were not conducted, and the amount of time students spent engaging with the educational materials differed across groups. Accordingly, the present study was designed to explore the potential effects of spaced repetition on learning rather than to isolate the individual effects of Kahoot and telesimulation on postpartum hemorrhage training. Future research may benefit from a more detailed evaluation of these educational tools by ensuring equal exposure time across groups, which would allow for clearer differentiation of the effects of specific learning strategies. In addition, although methodological precautions were implemented to minimize cross-group interaction, the possibility of informal peer communication outside the controlled online platforms cannot be entirely excluded due to the internet-based nature of the interventions.

## Conclusion

The study findings suggest that telesimulation and game-based Kahoot are effective learning methods for learning PPH management. This study, which focused on online educational interventions including telesimulation and game-based learning, warrants further development and evaluation in future research. Given the increase in the number of nursing students, the inadequacy of the number and equipment of simulation laboratories, and the limited access to simulation laboratories and faculties, it seems reasonable to expect that telesimulation and game-based programs will soon play an important role in nursing education. Using different methods in nursing education is effective and promising in developing students’ knowledge, skills, motivation, and clinical skills. While telesimulation demonstrated benefits in developing PPH knowledge and skills in this study, it was not compared to a highly accurate face-to-face simulation. Therefore, telesimulation is not an equivalent alternative, but rather an approach that can be used in situations where face-to-face simulation is not possible.

## Supplementary Information

Below is the link to the electronic supplementary material.

Supplementary Material 1

Supplementary Material 2

Supplementary Material 3

Supplementary Material 4

Supplementary Material 5

## Data Availability

The data generated and/or analyzed during the current study are available from the corresponding author on reasonable request.

## Abbreviations

PPH: Postpartum hemorrhage
CONSORT: Consolidated Standards of Reporting Trials
INACSL: International Nursing Association for Clinical Simulation and Learning
IMMS: İnstructional Materials Motivation Survey
SECPS: Self-Efficacy in Clinical Performance Scale
ANOVA: Analysis of Variance
