# Supplementary material for: Effects of game-based learning and telesimulation methods on the knowledge, skills, motivations, and self-efficacy levels of nursing students in postpartum hemorrhage management: a randomized controlled trial
Source: BMC Nurs. 2026 Jun 14;25:739. doi: 10.1186/s12912-026-04878-4 (PMC13491754; doi:10.1186/s12912-026-04878-4)
Supplement: Supplementary file 1 [file 12912_2026_4878_MOESM1_ESM.docx]

**İnterview questions with students**

- What factors did you consider when admitting the patient to the unit?
- Were you able to identify any early warning signs of bleeding? If so, what guided your assessment?
- How would you evaluate your communication within the healthcare team during the simulation?
- How did you communicate with the patient and her family, and what information did you prioritize sharing with them?
- Which clinical signs and findings did you focus on during the physical examination?
- How did you assess uterine tone and quantify the amount of bleeding?
- At what point did you recognize signs of hypovolemic shock?
- Which clinical findings indicated a deterioration in the patient’s condition?
- What interventions did you initiate to stabilize the patient?
- Which steps of the institutional or standardized postpartum hemorrhage protocol did you implement?
- How effectively did you follow the algorithm for managing PPH?
- What criteria did you use to determine the need for blood transfusion?
- How did you monitor the patient before, during, and after transfusion?
- What aspects of your performance during the simulation do you believe were most effective?
- Is there anything you would approach differently if given the opportunity?
