# Supplementary material for: Effects of game-based learning and telesimulation methods on the knowledge, skills, motivations, and self-efficacy levels of nursing students in postpartum hemorrhage management: a randomized controlled trial
Source: BMC Nurs. 2026 Jun 14;25:739. doi: 10.1186/s12912-026-04878-4 (PMC13491754; doi:10.1186/s12912-026-04878-4)
Supplement: Supplementary file 2 [file 12912_2026_4878_MOESM2_ESM.docx]

**PPH Information Form Question Examples**

1. Which of the following statements about uterine involution in the postpartum period is incorrect?
   a. Immediately after delivery, the fundus is below the umbilicus and rises to the level of the umbilicus after detachment of the placenta
   b. Two fingers above the umbilicus 12 hours after birth.
   c. Two fingers below the umbilicus on the first day after birth
   d. Four fingers below the umbilicus on the second day after birth
   e. The fundus is not palpated from the abdomen 10-12 days after delivery
2. Postpartum hemorrhage is classified based on causes, using the English acronym 4T. Which of the following options does not fall within the 4T?
   a. Tonus (Uterine atony)
   b. Tissue
   c. Tocolysis
   d. Thrombin (Coagulation disorder)
   e. Trauma
3. *Following a normal delivery, uterine muscle contractions prevent bleeding by compressing the open vascular endings at the site of placental separation. When the myometrium is not sufficiently contracted, the blood vessels are not compressed and bleeding occurs*.

Which of the following is an explanation for this occurrence?

a. Atony
b. Cervical laceration
c. Coagulation disorder
d. Placenta retention
e. Subinvolution of the uterus

4. What is the most common cause of PPH?
   a. Uterine atony
   b. Placenta retention
   c. Placenta previa
   d. Abruption of placenta
   e. Cervical laceration
5. When evaluating your patient at the 6th hour postpartum, what would you do as the first nursing action while making this evaluation?
   a. I would ask the mother to turn to my side
   b. I would ask the mother to lie on her back with her knees and legs straight
   c. I would ask the mother to urinate and empty her bladder
   d. I would ask the patient's relatives how much the patient bled after delivery and note it down
   e. I would help the patient with oral intake
6. What is the first-choice uterotonic agent to prevent postpartum hemorrhage?
   a. Prostaglandin F2a analog (Carboprost)
   b. Prostaglandin E1 analog. (Misoprostol)
   c. Oxytocin
   d. Ergot Derivatives (Ergometrine, Methylergonovine)
   e. Tranexamic Acid
7. On postpartum day 2, the mother presents with excessive vaginal bleeding, shallow and irregular breathing, tachycardic pulse, low blood pressure, and cyanosis of the lips and nails. Urine output decreased to <30 ml/h. What is the possible cause of this occurrence?
   a. Infection
   b. Uterine subinvolution
   c. Uterine inversion
   d. Hemorrhagic Shock
   e. Hematoma
8. Which condition is caused by factors such as attempting manual placenta removal before spontaneous detachment, preterm labor, and placental implantation anomalies that lead to postpartum hemorrhage?
   a. Placenta retention
   b. Reproductive organ laceration
   c. Uterine subinvolution
   d. Uterine inversion
   e. Hematoma
9. If a mother with postpartum hemorrhage has a blood pressure of 200/130 mmHg, which uterotonic drug is not used?
   a. Prostaglandin F2a analog (Carboprost)
   b. Prostaglandin E1 analog. (Misoprostol)
   c. Oxytocin
   d. Ergot Derivatives (Ergometrine, Methylergonovine)
   e. Tranexamic Acid
10. If the uterus is loose, diffuse and large when palpated and bleeding, multiple pregnancy and prolonged labor are present, what should be the primary consideration for this patient?
    a. Maternal fatigue
    b. Infection
    c. Hematoma
    d. Reproductive organ laceration
    e. Uterine Atony
