# Supplementary material for: Effects of game-based learning and telesimulation methods on the knowledge, skills, motivations, and self-efficacy levels of nursing students in postpartum hemorrhage management: a randomized controlled trial
Source: BMC Nurs. 2026 Jun 14;25:739. doi: 10.1186/s12912-026-04878-4 (PMC13491754; doi:10.1186/s12912-026-04878-4)
Supplement: Supplementary file 3 [file 12912_2026_4878_MOESM3_ESM.docx]

**POSTPARTUM HEMORRHAGE (PPH) TELESIMULATION SKILL EVALUATION FORM**

1) Patient admission and assessment

2) Communication

3) Physical examination

4) Signs and symptoms of hypovolemic shock

5) Postpartum hemorrhage protocol

6) Blood transfusion

| PATIENT ADMISSION AND ASSESSMENT |  | **Expressed**  **(1)** | **Did not express**  **(0)** |
| --- | --- | --- | --- |
|  |  |  |  |
|  | Expresses risk factors |  |  |
|  | Checks recent blood values |  |  |
| COMMUNICATION | Introduces himself-herself to the patient |  |  |
|  | Asks the patient how s/he feels |  |  |
|  | Evaluates behaviors and facial expressions of patients |  |  |
| PHYSICAL EXAMINATION | Controls uterine involution |  |  |
|  | Follows vital signs |  |  |
|  | It outlines the essential criteria to be considered during the examination of the skin and mucous membranes |  |  |
| SIGNS AND SYMPTOMS OF HYPOVOLEMIC SHOCK | Makes ABC control |  |  |
|  | Follows respiratory stress and oxygen saturation |  |  |
|  | Monitors dry mouth and feeling of thirst |  |  |
|  | Monitors symptoms of lethargy and weakness |  |  |
|  | Observes cold pale moist skin |  |  |
|  | Monitors weak and rapid pulse |  |  |
| POSTPARTUM HEMORRHAGE PROTOCOL | Controls bleeding |  |  |
|  | Calls for help |  |  |
|  | Monitors patients to follow vital signs |  |  |
|  | Examines the patient’s need for oxygen |  |  |
|  | Expresses that patient should be kept warm |  |  |
|  | Expresses the need for fundal massage of uterus |  |  |
|  | Evaluates the reason of bleeding. (4 T) |  |  |
|  | Expresses the need to empty the bladder by inserting a catheter |  |  |
|  | Measures the amount of bleeding |  |  |
|  | Expresses the need for emergency hemogram |  |  |
|  | Expresses the need to initiate intravenous fluid therapy |  |  |
|  | Expresses the need to initiate uterotonic drug treatment |  |  |
|  | Expresses the need to open a second vascular access |  |  |
| BLOOD TRANSFUSION | In accordance with the patient’s blood type, two units of erythrocytes are requested from the blood bank |  |  |
