# Supplementary material for: Effects of game-based learning and telesimulation methods on the knowledge, skills, motivations, and self-efficacy levels of nursing students in postpartum hemorrhage management: a randomized controlled trial
Source: BMC Nurs. 2026 Jun 14;25:739. doi: 10.1186/s12912-026-04878-4 (PMC13491754; doi:10.1186/s12912-026-04878-4)
Supplement: Supplementary file 4 [file 12912_2026_4878_MOESM4_ESM.docx]

## INSTRUCTİONAL MATERİALS MOTİVATİON SURVEY (IMMS)

**5. Very True, 4. True, 3. Moderately True, 2. Somewhat True, 1. Not True**

|  | **Instructional Materials Motivation Survey** **İtems** | **5** | **4** | **3** | **2** | **1** |
| --- | --- | --- | --- | --- | --- | --- |
| **1** | When I first saw this course, I thought it would be easy for me. |  |  |  |  |  |
| **2** | At the beginning of this lesson, there were some interesting things that interested me. |  |  |  |  |  |
| **3** | I understood this course material/tool (e.g. computer, book) more easily than I expected |  |  |  |  |  |
| **4** | After reading/listening to the introductory information, I was sure of what I needed to learn from this course. |  |  |  |  |  |
| **5** | Practicing this course gave me a sense of accomplishment. |  |  |  |  |  |
| **6** | It was clear to me how the content of this course related to what I had previously learned. |  |  |  |  |  |
| **7** | There was as much information as needed in the course material/tool. I was able to separate the things that were important. |  |  |  |  |  |
| **8** | The material/course tool used in this course was remarkable. |  |  |  |  |  |
| **9** | There were pictures, stories, or examples that showed how this course material/tool might be important to some people. |  |  |  |  |  |
| **10** | It was important for me to successfully complete this course. |  |  |  |  |  |
| **11** | The quality of the texts/voices made it easier for me to pay attention in class. |  |  |  |  |  |
| **12** | As I studied for this course, I became sure that I would learn the topics. |  |  |  |  |  |
| **13** | I enjoyed this class so much that I wanted to learn more about this topic. |  |  |  |  |  |
| **14** | The lectures in this material/course tool were enjoyable. |  |  |  |  |  |
| **15** | The content of this material/course tool was prepared according to the topics that interest me. |  |  |  |  |  |
| **16** | The way the information was organized in the course material made it easier for me to pay attention. |  |  |  |  |  |
| **17** | The course material included examples and explanations of how people use the information in this course. |  |  |  |  |  |
| **18** | The exercises in this lesson were very easy. |  |  |  |  |  |
| **19** | There were some intriguing things in this lesson. |  |  |  |  |  |
| **20** | I really enjoyed studying this course |  |  |  |  |  |
| **21** | The number of topic repetitions in this course was sufficient. I was never bored. |  |  |  |  |  |
| **22** | The information in this course and the way the information was presented made me think that this course was important. |  |  |  |  |  |
| **23** | I learned unexpected or surprising and useful information in this course. |  |  |  |  |  |
| **24** | After studying this course for a while, I was sure that I would be successful in this course. |  |  |  |  |  |
| **25** | The feedback (feedback/comments) after the exercises made me feel like my work was paying off. |  |  |  |  |  |
| **26** | Various elements such as reading passages, exercises, pictures, videos, and sounds helped me pay attention to the lesson. |  |  |  |  |  |
| **27** | The style of the writings in the course material was suitable for me. |  |  |  |  |  |
| **28** | I was able to relate the content of this course to things in my own life |  |  |  |  |  |
| **29** | Completing this course successfully made me feel good. |  |  |  |  |  |
| **30** | I believed that the content of this course would be useful for me. |  |  |  |  |  |
| **31** | I understand all parts of this course material |  |  |  |  |  |
| **32** | The course material content was well prepared. Therefore, my confidence that I will be successful in the course has increased. |  |  |  |  |  |
| **33** | The lesson was prepared so well that it was a pleasure for me to teach this lesson. |  |  |  |  |  |
