# Supplementary material for: Effects of game-based learning and telesimulation methods on the knowledge, skills, motivations, and self-efficacy levels of nursing students in postpartum hemorrhage management: a randomized controlled trial
Source: BMC Nurs. 2026 Jun 14;25:739. doi: 10.1186/s12912-026-04878-4 (PMC13491754; doi:10.1186/s12912-026-04878-4)
Supplement: Supplementary file 5 [file 12912_2026_4878_MOESM5_ESM.docx]

**SELF-EFFİCACY SCALE in CLİNİCAL PERFORMANCE**

| **than I can do in clinical practice.** | **I am not sure**  **I'm Totally Sure** | | | | | | | | | | |
| --- | --- | --- | --- | --- | --- | --- | --- | --- | --- | --- | --- |
|  | **%0** | **%10** | **%20** | **%30** | **%40** | **%50** | **%60** | **%70** | **%80** | **%90** | **%100** |
| 1. I can collect important data through physical diagnostics |  |  |  |  |  |  |  |  |  |  |  |
| 2. I can collect data about the patient by taking the patient's history. |  |  |  |  |  |  |  |  |  |  |  |
| 3. I can collect data about the patient by motivating myself and the patient. |  |  |  |  |  |  |  |  |  |  |  |
| 4. I can manage time appropriately when collecting data. |  |  |  |  |  |  |  |  |  |  |  |
| 5. I can collect objective data about the patient's health status. |  |  |  |  |  |  |  |  |  |  |  |
| 6. I can collect subjective data about the patient's health status. |  |  |  |  |  |  |  |  |  |  |  |
| 7. I can correlate information obtained from different sources about the patient. |  |  |  |  |  |  |  |  |  |  |  |
| 8. I can save the data I collect about the patient's health status. |  |  |  |  |  |  |  |  |  |  |  |
| 9. I can analyze the data collected about the patient's health status |  |  |  |  |  |  |  |  |  |  |  |
| 10. I can identify the patient's strengths throughout the care process. |  |  |  |  |  |  |  |  |  |  |  |
| 11. I can identify the patient's health concerns throughout the care process. |  |  |  |  |  |  |  |  |  |  |  |
| 12. I can prioritize the patient's health-related problems |  |  |  |  |  |  |  |  |  |  |  |
| 13. I can express nursing diagnoses precisely and clearly based on the data collected. |  |  |  |  |  |  |  |  |  |  |  |
| 14. I can clearly and precisely express nursing diagnoses regarding the factors that cause health problems in the patient. |  |  |  |  |  |  |  |  |  |  |  |
| 15. I can list nursing diagnoses according to established priorities. |  |  |  |  |  |  |  |  |  |  |  |
| 16. I can clearly and precisely express all goals in the patient's care plan. |  |  |  |  |  |  |  |  |  |  |  |
| 17. I can clearly and precisely express the short-term goals in the patient's care plan. |  |  |  |  |  |  |  |  |  |  |  |
| 18. I can clearly and precisely express the long-term goals in the patient's care plan. |  |  |  |  |  |  |  |  |  |  |  |
| 19. I can clearly and precisely express measurable results for goals. |  |  |  |  |  |  |  |  |  |  |  |
| 20. I can develop the patient's daily care plan in line with the goals. |  |  |  |  |  |  |  |  |  |  |  |
| 21. I can develop the patient's daily care plan according to the determined priorities. |  |  |  |  |  |  |  |  |  |  |  |
| 22. I can follow instructions in the patient care plan to achieve set goals. |  |  |  |  |  |  |  |  |  |  |  |
| 23. I can provide care to the patient in line with the determined priorities. |  |  |  |  |  |  |  |  |  |  |  |
| 24. I can implement a patient care plan in line with available resources. |  |  |  |  |  |  |  |  |  |  |  |
| 25. I can explain the procedure to the patient and family members before implementing each nursing intervention. |  |  |  |  |  |  |  |  |  |  |  |
| 26. I can collaborate with the patient or family members in implementing the daily care plan. |  |  |  |  |  |  |  |  |  |  |  |
| 27. When I encounter a situation similar to my previous experiences, I can make a decision based on my experiences. |  |  |  |  |  |  |  |  |  |  |  |
| 28. In difficult situations, I can ask for help from a mentor/counselor or other healthcare personnel |  |  |  |  |  |  |  |  |  |  |  |
| 29. I can improve my skills in line with the feedback from my mentor/consultant or other healthcare professionals. |  |  |  |  |  |  |  |  |  |  |  |
| 30. I can prepare a discharge plan for the patient. |  |  |  |  |  |  |  |  |  |  |  |
| 31. I can record and report daily clinical work. |  |  |  |  |  |  |  |  |  |  |  |
| 32. I can evaluate whether the results set for the patient have been achieved.. |  |  |  |  |  |  |  |  |  |  |  |
| 33. I can evaluate how nursing interventions are implemented/carried out. |  |  |  |  |  |  |  |  |  |  |  |
| 34. I can identify points that are not working or are problematic from the steps of the nursing process. |  |  |  |  |  |  |  |  |  |  |  |
| 35. I can decide whether to change or continue the care plan based on the patient's prognosis. |  |  |  |  |  |  |  |  |  |  |  |
| 36. I can make decisions about varying outcomes based on the patient's prognosis. |  |  |  |  |  |  |  |  |  |  |  |
| 37. I can determine priorities in line with the patient's prognosis. |  |  |  |  |  |  |  |  |  |  |  |
